# Co-Designing an Integrated Care Network with People Living with Parkinson’s Disease: A Heterogeneous Social Network of People, Resources and Technologies

**DOI:** 10.3390/jpm12061001

**Published:** 2022-06-19

**Authors:** Amélie Gauthier-Beaupré, Emely Poitras, Sylvie Grosjean, Tiago A. Mestre, on behalf of the iCARE-PD Consortium

**Affiliations:** 1 Faculty of Health Sciences, University of Ottawa, Ottawa, ON K1N 6N5, Canada; agaut039@uottawa.ca; 2 Department of Communication, University of Ottawa, Ottawa, ON K1N 6N5, Canada; epoit093@uottawa.ca; 3 Parkinson Disease and Movement Disorders Centre, Division of Neurology, Department of Medicine, The Ottawa Hospital Research Institute, University of Ottawa Brain and Mind Research Institute, Ottawa, ON K1Y 4E9, Canada; tmestre@toh.ca

**Keywords:** Parkinson’s disease, integrated care network, co-design, actor-network theory, qualitative research, eHealth technologies, care partners, health care professionals

## Abstract

As part of the iCARE-PD project, a multinational and multidisciplinary research endeavour to address complex care in Parkinson’s disease, a Canadian case study focused on gaining a better understanding of people living with Parkinson’s disease (PwP) experiences with health and medical services, particularly their vision for a sustainable, tailored and integrated care delivery network. The multifaceted nature of the condition means that PwP must continuously adapt and adjust to every aspect of their lives, and progressively rely on support from care partners (CP) and various health care professionals (HCP). To envision the integrated care delivery network from the perspective of PwP, the study consisted of designing scenarios for an integrated care delivery network with patients, their CP and their HCP, as well as identifying key requirements for designing an integrated care delivery network. The results demonstrate that numerous networks interact, representing specific inscriptions, actors and mediators who meet at specific crossing points. This resulted in the creation of a roadmap and toolkit that takes into consideration the unique challenges faced by PwP, and the necessity for an integrated care delivery network that can be personalized and malleable so as to adapt to evolving and changing needs over time.

## 1. Introduction

Parkinson’s disease (PD), a progressive neurodegenerative condition, impacts the lives of those living with the condition and their loved ones in various areas of daily life. The complex, unpredictable and fluctuating nature of the condition means that people living with Parkinson’s disease (PwP) must adapt and adjust every aspect of their lives continuously. With needs that become increasingly complex to manage, PwP rely heavily on support from care partners (CP) and various health care professionals (HCP). It is not rare to see a variety of HCP be involved and consulted to support the management of the condition. These may include medical specialists, allied health professionals and other community resources. While there is a clear need for multispecialty care in PD, a sustainable model of multidisciplinary PD management is lacking [1,2,3]. Currently, models of care have many drawbacks, such as a lack of multidisciplinary collaboration, a lack of access to care delivery at home or in the community, and a failure to take the social needs of patients and families into account [4,5,6,7].

To address these gaps, the focus should be placed on co-designing a sustainable, tailored and integrated care network with PwP. Using the Actor-Network Theory [8,9], we understand that the care for PwP takes place in a heterogeneous social network of human, textual and material actors, such as people, technologies and resources, that connect together with associations and alliances [10,11]. As such, an integrated care network should leverage all necessary sectors in a coherent and effective manner to ensure patient care is tailored to the multidimensional needs of PwP [12]. As highlighted in the World Health Organization’s global strategy on people-centred and integrated health services, integrated care considers various aspects of care delivery “in a way that ensures people receive a continuum of health promotion, disease prevention, diagnosis, treatment, disease management, rehabilitation, and palliative care services, at different levels and sites of care within the health system, and according to their needs throughout the life course” [13] (p. 7).

Considering that PwP must navigate through a continuum of care services that is always evolving, integrated health services that combine a plethora of heterogeneous elements become a critical part of their journeys. In addition, the unique challenges faced by PwP emphasize the need for integrated care that can be personalized and malleable to evolving and changing needs over time. To ensure this integrated care network is oriented towards the PwP, there must be “a coherent set of methods and models on the funding, administrative, organizational, service delivery and clinical levels designed to create connectivity, alignment, and collaboration within and between the cure and care sectors” [14] (p. 3). The design of such methods and models to optimize care for PwP necessitates a bottom-up approach, leading to concrete patient-centred solutions.

Through a co-design approach, patients’ experiences are at the centre of the design process [15,16]. For PwP, this approach is extremely relevant as it gives them the opportunity to play a critical and pivotal role in envisioning solutions that meet their needs for an integrated care network. As the main beneficiaries of this network, PwP should be able to offer insights, perspectives and experiences in its design.

The aim of this article is to provide a better understanding of experiences with health and medical services and to envision the integrated care delivery network from the perspective of PwP, their CP and HCP. We will present the results from participatory design workshops held in Canada with PwP, their CP, and HCP in the context of the iCARE-PD project. The analysis of the workshops allowed us to identify limitations in the current care network where coordination of care relies heavily on patients. We will also identify desires and requirements for a sustainable integrated care network that is personalized, and meets the evolving needs of PwP. The results of this study can inform the development of sustainable integrated care networks to better equip PwP to live fulfilling lives.

## 2. Materials and Methods

### 2.1. Context of the Study: The iCARE-PD Project

**Figure 1:**
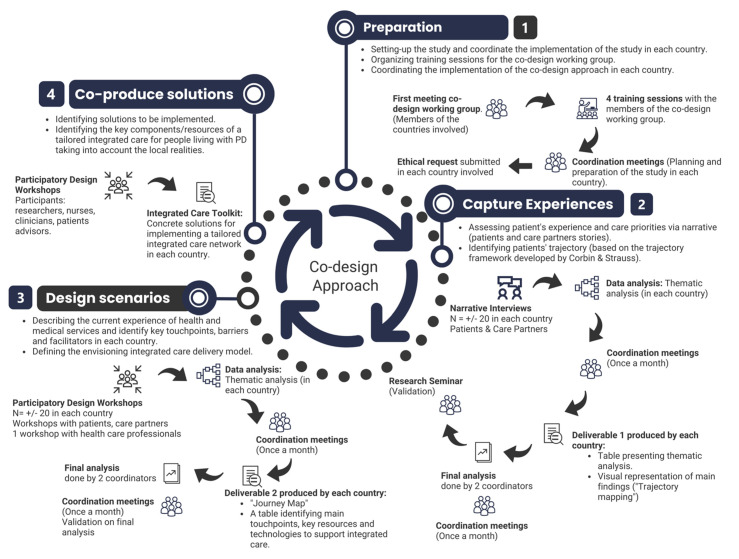
Co-design approach for the iCARE-PD project.

In response to the complex situation faced by PwP, a group of neurologists have developed the project on integrated Parkinson care networks: addressing complex care in Parkinson disease in contemporary society (iCARE-PD) [17]. This multidisciplinary and international study uses a co-design approach to develop a home-based health care delivery model based on integrated care, self-management support and technology-enabled care [18]. We used a co-design approach largely informed by the field of participatory design and Experience-Based Co-Design (EBCD) where the patients’ perspective is seen as a central component to the design process [19,20]. Our approach consisted of four linked steps that were coordinated among five countries: (1) Preparation (2) Capture patients’ experiences by using narrative interviews and understand the patients’ trajectory; (3) Design with patients, CP, and HCP scenarios for an integrated care delivery network and (4) Co-produce solutions by identifying key requirements for designing an integrated care network (Figure 1). Each country conducted the research activities and shared their results with the larger group for a final analysis.

Within the context of the iCARE-PD project, this article relates to the findings from the third phase, the “design scenarios” part of the four-phased, co-design approach. In the spirit of patient-centred care, ‘patients as partners’ are invited in the early stages of the design process to share their experiences in managing their medical condition. The aim of our co-design approach was not only to elicit contextual information or describe patient experiences, but also to sustain the collaborative construction of new interpretations and enable various stakeholders to ‘make sense together’ and co-produce knowledge [21].

For this article, we will be sharing the results from the Canadian study specifically. While the first two phases of the approach helped prepare the groundwork for the collective effort to capture patients’ and CP’ stories to identify care trajectories [22,23], it is in this third phase that respondents participate in design workshops to help develop an improved model of integrated care. At this stage of the research project, each country developed a “journey map” and a table identifying main touchpoints (an important point of contact with a person or service at the time of the event), key resources, and technologies to support integrated care. This step will then lead to the fourth and final stage, which is co-producing solutions for implementing a tailored, integrated care network in each country with recommendations as deliverables.

### 2.2. Sampling

Purposive sampling was used to select participants for phase 3 “design scenarios” of the co-design approach. Sampling criteria were developed in collaboration with our tertiary PD centre to ensure adequate diversity in the sampling in terms of gender (women, men and other genders), PD stage (stages 1–5), age (all ages) and years since diagnosis (Table 1). The PD stage is determined mostly by motor symptoms and self-care abilities. On this scale, 1 and 2 reflect early-stage PD, 2 and 3 represent mid-stage PD, and 4 and 5 represent advanced-stage PD. With respect to recruitment, participants were approached by neurologists and PD staff in a hospital in Canada. The invitation to participate provided participants with information about the study and noted that participation would involve discussion among focus groups with a maximum of six participants per group. Participants were assured that their data would be treated confidentially, and written and oral informed consent were obtained prior to data collection. The study was approved by the Research Ethics Boards at the Ottawa Hospital Research Institute (Protocol # 20180561-01H) and the University of Ottawa (#S-11-18-472).

### 2.3. Data Collection

Owing to the COVID-19 pandemic, the participatory design workshops were conducted remotely between June and October 2021. The participants received by mail clear instructions and a package for a written exercise to be completed at home prior to the workshop. Participants had the option to complete the exercise alone or with the help of a CP. For the workshop, the participants also had the option to join on their own or with a CP.

#### 2.3.1. Pre-Workshop Tasks for PwP and Their CP

Prior to the workshops with PwP and CP, the team provided participants with 2 activities which would later be used to guide the discussions. The first activity, a journey mapping exercise, asked participants to describe their current experiences with health and medical services by identifying key touchpoints, barriers, and facilitators to accessing and using these services (Figure S1). Participants were provided with a journey map template including predefined event cards and emotion stickers ahead of the workshop. They were asked to identify 2 or 3 major changes or events (medical, social, emotional, physical, professional, etc.) in their journey with PD and identify the key related touchpoints (people, community resources, health/social services), their experiences with that event and barriers and facilitators that have influenced their experience with that event. They were asked to write that information in the journey map template so it could be used to guide their reflections during the workshop.

The second activity, inspiration cards, asked participants to create a scenario of their vision for an integrated care delivery model (home and community care) that puts PwP at the centre of care delivery (Figure S2). Again, participants were provided with an inspiration card template along with a set of predefined cards from which they could select to include in their vision. They were asked to select 3 or 4 cards for each of the categories identified in the template. Red cards were “interaction cards” to identify interactions with key people or services facilitating linkages, green cards were “resource cards” to represent helpful resources to manage PD over time, and black cards were “technology cards” for technologies to support at home/community models of care delivery.

#### 2.3.2. Workshop for PwP and Their CP

Participants were invited to take part in an online, 45-min workshop in the form of a focus group to discuss their answers to the 2 activities that they completed at home. For participants that could not attend the focus group sessions, separate semi-structured interviews were organized between the researchers and participants. The purpose of the workshop was to determine PwP’s and CP’s priorities in terms of care delivery based at home or in the community from the perspective of PwP. For activity 1, participants were asked to report with a higher level of detail on one or several of the events they have identified in their journey maps. For activity 2, participants reported on and explained their selection for specific cards they had selected in their templates. For the workshops that were held in a focus group, participants were asked to share their insights for both activities on a roll-call basis to ensure all participants had the opportunity to speak.

#### 2.3.3. Pre-Workshop and Workshop with HCP

For HCP participating in the study, activities 1 and 2 were similar to those of PwP and CP. However, for activity 1, HCP were asked to complete an online questionnaire which contained instructions for this self-guided component, while no pre-workshop tasks were assigned for activity 2 which would be completed as a group during the workshop. To target and help define the pre-workshop questionnaire for activity 1, participants were presented with two personas, meaning two patient types within a targeted demographic, disease stage, attitude or behaviour. The first persona represented a 59-year-old woman who was recently diagnosed and experienced symptoms such as fatigue while working as an elementary school teacher. The second persona was of a 78-year-old man in a rural area living with an advanced stage of the disease while managing personal goals such as continuing physiotherapy (Figure S3). Through questionnaires, HCP were asked to identify two or three changes (positive or negative) or medical/social episodes that take place during the patient journey for both personas. For each event in a persona’s patient journey, HCP were also prompted to identify and describe key touchpoints, helpful resources and barriers, as well as emotions they think patients would be experiencing. During the workshop, HCP shared their answers to the online questionnaire with other participants. For Activity 2, the workshop moderator led the group to brainstorm about how they envision an integrated care model for PwP. To facilitate the discussion, an online collaborative platform (Miro online whiteboard) displayed the series of red, green and black inspiration cards (Figure S4). Participants discussed their thoughts and collaboratively selected inspiration cards, based on the two personas as well as their own professional experiences.

### 2.4. Data Analysis

Prior to data analysis, recordings from the workshops were transcribed verbatim and organized in the qualitative data analysis software (QSR International NVivo 12).

Thematic analysis was used to identify, analyse and report on the patterns that were identified in the transcriptions of the workshops for activity 1—journey map [24], as well as highlighted important events and envisions for integrated care networks in activity 2—envisioning cards. The anonymized transcripts were coded independently by two members of the research team using an inductive-deductive approach [25]. As such, the deductive reasoning was used by selecting core themes from the key concepts of the ANT and supplementing the codes using an inductive reasoning where important moments were recognized in the transcripts and added to the predefined coding structure. First, anonymization of transcripts was done by assigning pseudonyms to participants: P1-P10 for patients; CP1-CP3 for CP; and HCP1-HCP5 for HCP, and by removing any identifiable information within the transcripts. Subsequently, two members of the research team reviewed the transcripts to code segments based on a predetermined coding structure. Inductive coding was used to identify new codes, and consensus was obtained during a meeting with the research team. For Activity 1 (Journey Map), the coding structure included 4 domains: (1) events (episodes of social and medical care selected by PwP [Storyline]), (2) touchpoints for each event, (3) barriers to making community linkages, to using health care services or community resources, and (4) facilitators to making community linkages, to using health care services or community resources. For activity 2 (envisioning cards), the coding structure included 3 domains: (1) resources, (2) interactions, and (3) technology. Subsequently, the research team created a mind map to better represent patterns visually for both activities. This allowed the researchers to reach a higher level of abstraction and identify key themes of the participatory design workshops for both the journey map and visions for an integrated care network.

Lastly, the researchers conducted a final analysis for activity 2 using the key concepts of the Actor-Network Theory or ANT [8,9,26,27]. This analysis allowed for a deeper understanding of the heterogeneous social network of actors that connect together to create an integrated care network [28]. The ANT is a sociological and anthropological approach used in organization and communication studies, and its key concepts are used to understand how actor networks emerge, how they are composed and constituted, and how they are maintained over time (Table 2).

In our study, we have used ANT to examine the nature of the integrated care network and its components co-created with PwP, CP and HCP. By studying the integrated care network as an actor-network, composed of a plethora of heterogeneous elements (such as nurses, informal caregivers, technologies, rules, skills, management tools, etc.), we can align support provided to PwP in their daily life by offering personalized and tailored care. In the context of our study, we explore the organization of local networks and their reconfiguration over time to support medical and social care at home. For example, they identified intermediaries or mediators who can form relationships between various actors to support care delivery at home.

## 3. Results

In this section, the results are presented in two parts to highlight the main findings at the end of each activity. First, we describe (by using a journey map visualization) the current experience of medical and social services from the perspective of PwP, CP and HCP by identifying key events, touchpoints, barriers and facilitators. Second, we present the key elements or components of an integrated care network co-produced by the participants during the workshop using the key concepts of the ANT framework.

### 3.1. Activity 1: Exploring with PwP & HCP the Actual Network of Care

**Figure 2:**
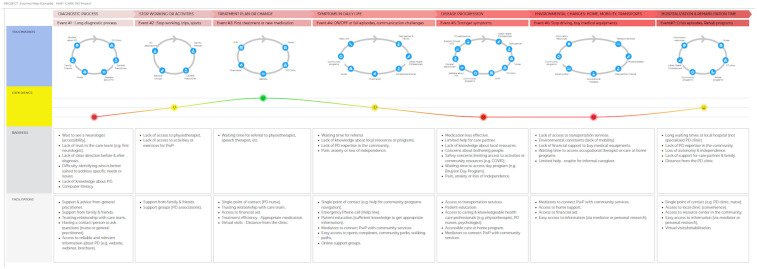
Journey Map for Canada.

The purpose of this activity was to create a map of patients’ journey from diagnosis to daily life with PD. Journey maps are used to depict the current experience of care models from the perspective of patients [30,31]. For Activity 1, thematic analysis was used to identify themes and subthemes (Table 3). The main findings were summarized in a visual representation called the “journey map” (Figure 2). The Journey map provides a visual representation of the current state of care delivery in Canada by identifying: (a) key events in the storyline of the PwP, (b) important touchpoints, (c) experiences and feelings and (d) barriers/facilitators to making community linkages, as well as to using health care services or community resources.

Thematic analysis revealed seven key events that represent significant changes either medically or socially for which they need support or access to medical or social services (Table 3). For each event, participants identified barriers and facilitators to accessing care or resources.

#### 3.1.1. Process of Diagnosis and Diagnosis Announcement

For many patients, diagnosis was found to be a key moment after waiting for a long period of time. Often, patients knew ahead of diagnosis that something was wrong and that they should seek medical attention. When navigating the health care system, there seem to be mixed experiences with being able to access specialists and get referred to a specialized clinic. Some were able to get the attention from a specialist and a referral to a clinic rapidly while others had more difficulty doing so.

*P5: Well, I think, of course the main event was the diagnosis, […], which was three years ago. But I think I knew, like I knew something was wrong before that, I didn’t know… I had a lot of pain, but I was almost relieved when, I should say, I just, once I knew what the diagnosis was, and I knew I would learn how to cope with it somehow.*

For some people with greater difficulty accessing specialized care, a key factor is related to their identity. For women, there seemed to be greater challenges in getting the PD diagnosis because they felt that they were not being heard when their first symptoms began. Their experiences were diminished and not acknowledged by some HCP.

*P8: to get the diagnosis, it took a while. But I mean that’s not, I know it’s hard to diagnose Parkinson’s, and because I don’t have a family history for it, because I was female, I was young. The typical symptoms, I didn’t fit everything. And, I think it was just my situation. But also, not being taken seriously [by] the two of the neurologists. And that’s a barrier, but I think that, like I said, I think that’s a lot of women in this country.*

When diagnosis was reached by patients, the process of obtaining it was facilitated by a key touchpoint, the neurology clinics. For patients that had access to those clinics, they identified them as central to the diagnosis announcement. Patients felt like the care teams in the neurology clinics had the necessary knowledge and expertise to pose the diagnosis and provide support throughout that process.

*P5: And I certainly have the great treatment and people looking after me for all my Parkinson’s symptoms, so the touchpoints for me were, of course, the first touchpoint was the neurological clinic.*

Following diagnosis, knowledge of the disease and clear directions and instructions on managing the disease are critical. For PwP in Canada, this was identified as a clear barrier in their journey. Sometimes, patients mentioned being left alone with no clear direction and instructions on what to do and where to go next. This was also associated with feelings of anxiety and uncertainty for many.

*P1: Okay, well I can tell you that I was not geared or pointed towards any community services or any information of any kind for Parkinson’s.*

As a result, PwP used a lot of their own channels to find information about PD. For example, patients mentioned joining information and support groups to get the necessary information. Others also mentioned using their own research skills to navigate the wealth of information they could find related to PD. The endeavour of joining support groups and conducting their own research was mostly associated with positive experiences where they felt pride in being able to access information about PD.

*P1: I finally found what I found by myself, and I didn’t find any support, actually. The support was very poor, I have to admit, with everything you hear on the news, and anything else, about the research and everything else about Parkinson’s.*

*P8: I believe that, even when I go to the clinic, you know, you’re just giv[en] a sheet saying this is the symptoms. Like […], there’s no place to direct you, and you have to do your homework on your own. I am, I don’t know how other people find that, but that’s been my, my experience.*

*P6: Yeah then the same thing with the group that started that in winter. And we joined the group there, the Parkinson’s group, and it was just tremendous. And that’s when we got most of our information, because when he was diagnosed, they just left us on our own, and we were just, you know.*

With many obtaining knowledge and information from their personal networks and through their own means, it pointed to clear gaps in terms of an organized and supportive network of information and resources. Unfortunately, it is not all patients that were well surrounded and supported to obtain information. Some had limited personal support networks, so for those that were more isolated, the risk of facing greater inequalities in receiving supportive and quality follow-up increased.

Following diagnosis, the main supporting entity for PwP were family and friends. They were seen as playing an essential role early on after diagnosis since they were helpful in looking for information and resources, and for providing emotional support. Once more, personal support networks were starred and seen as central for PwP to learn to live with their new diagnosis of PD.

*P2: One thing I did do after the diagnosis, but not too long after, was going to a physiotherapist who was recommended by a friend of mine to do some, well a series of exercises which she’d be trained on.*

However, personal support networks have their limits, and the need for formal guidance and support services is critical. The lack, thereof, is problematic as it forces PwP to rely on their personal support networks for specific and complex guidance. Those with limited support in their personal lives may face greater social isolation and incomprehension on what to do following the PD diagnosis.

While there is a strong reliance on personal networks, PwP also emphasize the role of general practitioners in supporting their diagnosis with PD. General practitioners’ lack of expertise and knowledge about PD and the geriatric population more generally is a true impediment to serving those individuals. In addition, their limited communication with the PD care team limits their ability to have a role in this support function. PwP feel that general practitioners are easily accessible and therefore wish that they would be better equipped to respond to their immediate needs or refer them to appropriate pieces of information or resources. Neurologists often had to fill in for the missed opportunities of general practitioners to care for their patients with PD.

*HCP2: Yeah, I mean, I think one of our main struggles is how […] we can be appealing for general practitioners to care for people with Parkinson’s. That’s a barrier, but in a sense, […] could be a facilitator. […] But definitely, I think both P5 and P1, you know, we end up seeing ourselves being the GPs of our patients, which also shouldn’t happen, so how you strike this balance and make family doctors more at ease of managing problems that do happen let’s say in the geriatric population, but not necessarily specific of Parkinson’s, and so, in a way, but they could also manage those problems, for example.*

Thinking about the context of Canada, the role of general practitioners is essential in ensuring this kind of support for PwP and geriatric patients. More specifically, this role is amplified in rural and remote locations where specialists and neurologists may not always be present and accessible. General practitioners serve to bridge the gap that may exist between people living in rural and remote locations and specialists, therefore it is critical that they have appropriate knowledge and sufficient expertise in PD.

#### 3.1.2. Impact of PD on Work Life, Social Activities and Trips

PD impacts many aspects of daily life for those that receive the PD diagnosis. One major area that is impacted is people’s professional lives. Patients expressed that they were conscious that their disease would have an impact on their professional and social lives and that they would have to adapt to their new reality. The difficult part for many was that they would have to deal with those changes on their own and receive little to no support in this transition. For some, this change happened at a moment when they were not expecting it, leaving them with negative experiences or having to find adaptations on their own. These fears were expressed by both CP and PwP themselves.

*HCP2: I think I mentioned what would happen if she would change schools for example and she would practice without a place. That was one aspect and then um sure I’m you want to talk about barriers, facilitators but that was something that occurred to me in terms of a teacher that could all of a sudden have to teach in another school, another place, and so how that would change her care, that was one.*

*P3: The idea is I ended up stopping working voluntarily because I was losing power on one side, and I was doing physical work, before somebody gets hurt, I decided to take myself out of the job.*

Knowing that changes in the professional realm would occur, PwP need to have access to resources, to information to help them better understand the disease and to better comprehend the impacts it may have on their professional careers. Help is needed to allow PwP move beyond the acceptance of changes in their professional lives to the understanding that adaptations are possible to help them continue to live meaningful professional lives.

*P5: I think, you know, people faced with early on in their Parkinson’s they wanna just, you know, they the initial reaction I think a lot of people have is just keep plugging away and keep everything as normal as possible but is that really in their best interest, but helping them work through that problem, there’s definitely a lack of resources, I think, to help them work through that specific issue.*

HCP also reflect on this issue by acknowledging the need to investigate deeper into explanations for certain symptoms to support PwP to continue with their active lives longer.

*HCP4: One of the things I’m truly interested in if she’s very young, she has a full-time job, very demanding in elementary school. So, for me, education really with respect to why is she having such terrible fatigue because we really want to happen to her, with how young she is, is to keep her active and keep her functioning higher for longer. So, for me, education is really key with understanding what is going on in the sleep. What’s going on with fatigue, can we better manage that to actually address potential issues as well when people aren’t sleeping well and that increases their risk for having, you know, other problems and that emotional field. So, for me it’s the education on that level is where I would like to see some input.*

This educational component was important professionally for PwP but also in all aspects of their lives. They have indicated that being proactive (e.g., being able to gain knowledge about PD, being involved in a PD association and trying new activities) was identified as a facilitator to overcoming challenges and transitions in their journey with PD.

However, fragmentation of community services remains problematic and was identified as a major barrier for PwP. In fact, much of the responsibility to identify the resources available is left in the hands of patients. Some have the energy and capabilities to identify the resources available to them, but this may not be feasible for others. This can unfortunately widen the inequalities in access to care depending on patients’ circumstances. Thinking about social determinants of health, several contextual and identity factors may intersect to amplify these inequities that exist between the diverse PwP. CP specifically spoke about the need to have increased direction on available community services for PwP.

*CP3: Yeah and to be honest, P3 isn’t at a point with the illness where we’ve done much in this, along these lines, but I would imagine that it would be great if there was a focal point somewhere and you can go and you can ask “well, what physiotherapy practices in [name of city], specialized in…?”. We found that out through word of mouth. What about speech? I don’t know if some Parkinson’s patients that want to access speech patho or speech therapist, massage therapist, like it’s all, at least it has been for us, and maybe we haven’t found the key yet, the keyhole in the door. But as we try to find those supports, we kind of starting all over all the time. And we ask a friend like a physio “Who do you know that has specialized in Parkinson’s?”. And then you go to the physio you and “what about massage therapy and who do you know?” [P2 nods]. It’s just not as connected as it should be in my view.*

#### 3.1.3. Treatment Plan or Changes in the Treatment

Throughout the journey with PD, an adequate treatment plan is important to allow PwP to continue with their daily activities. Ensuring that a treatment plan is effective and ensuring a reduction of symptoms are just two examples of things that can give hope to PwP to continue living with a certain level of quality of life. Medication specifically was mentioned by patients as being a significant event in their journeys.

In this regard, communication with the medical team on treatment is vital and seen as a facilitating factor. Patients noted that treatment plans need to take a personalized approach to ensure that their needs are addressed.

*P7: Oh, I think the first one would have been appropriate medication and treatment for my Parkinson’s symptoms, which made a huge difference […] I was able to do more physical and social activities once I got on the medication. I kind of didn’t want to do it for quite some time. I thought I could manage it myself but it just came to a point where I realized that I did need some extra help. So that made a huge difference for me.*

Treatment plans that include home supports were valued by many patients and HCP. However, there was a consensus that home supports were constrained by financial burdens. The provision of community supports was said to be financially demanding for community organizations while equipment to promote independent living were considered monetarily out of reach for PwP living in the community.

*HCP survey response: LHIN [Local Health Integration Network] is limited in what they can provide due to budget cuts and can’t offer the home health support they would benefit from. The cost of equipment in the home is high and they may not be able to afford all that is suggested to them to improve quality of life and prevent falls.*

As PwP are learning to live with their condition, treatment plans and adaptations to treatment plans make a huge difference in their ability to have a good quality of life. While attentive communication with the health care team is essential, barriers to financing can impede the successful delivery of effective supports.

#### 3.1.4. ON/OFF Episodes or Communication Challenges

Another significant event in the journey with PD is adapting to changes and episodic symptoms. Patients discussed their issues with communication, for example, which impacts their daily activities and social lives. Some of these episodic symptoms and changes to their capabilities necessitate support and help, which are often very hard for them to find. This is especially hard when they require specialized help.

*P8: The other one I did was, like, my progression with Parkinson’s has been a little hard, especially […] ‘cause I’m having communication problems as you can tell [chuckles], so I’m trying to find the right words and that, and my speech has changed. It’s a little lower. Those are the challenges. The communication has been a big change in my new symptoms.*

As was mentioned previously, access to help can be challenging for many. Accessing the right people but also accessing the support in a timely fashion can be very difficult. This can have devastating consequences and negatively impact PwP’s ability to have a good quality of life.

*P8: Well, I’m still waiting […] for a speech therapist referral. It’s been since April so I figured I’m gonna call back the clinic, to figure out why I haven’t gotten one yet.*

Patients also brought our attention to a lack of expertise from HCP like physiotherapists, among others. This challenge is amplified for PwP living in rural or remote locations. Well documented is the limited capacity for rural, remote and smaller locations in Canada to host specialized medical services. Virtual consultations have been suggested to counteract this gap for those regions.

*P5: one of the barriers that immediately comes up is small-town Canada and how people in Canada access specialized services so I think that really, that unfortunately in a lot of places, that’s just not possible because there aren’t any specialized services in the smaller towns and especially if you’re thinking about, you know, physiotherapy or those types of things where the local physiotherapist might not have seen very many Parkinson’s patients, but I think that the idea that maybe the most practical for them is to try to access local, whatever the resources are locally, even if they’re not super specialized.*

Access to staff with PD expertise in the community was identified as a barrier by PwP. Services developed specifically for people with PD such as physiotherapy, exercise programs, or speech therapy led by allied health professionals with expertise in PD were identified as rare in the community. Telehealth such as virtual consultations have been suggested by some participants to overcome this shortage of expertise. This solution would be appropriate and fit with the geographic specificities in Canada.

#### 3.1.5. Stronger Symptoms, Access to Home Care or Medical Equipment

The challenges amplify as PwP experience stronger symptoms over time. In preparation for this transition, patients expressed their outlook in anticipating their future needs as they began looking into community resources for the required support. Particularly, both CP and PwP themselves were beginning to plan for home care support services. Many of them shared a desire to be accompanied in the planning of progressive PD care, such as being provided with the necessary medical equipment. They explained they needed to be guided as they searched and selected resources within the community in anticipation of the impact of PD on daily life. In addition, HCP also recognized this need for PwP to be guided when navigating the health care system.

*P3: Specifically, here is to start thinking about an assisted living-like situation. How long are they going to be in their home? How long are they going to be able to negotiate stairs, etc. […]. So with falls or near falls that have happened. There’s only so much that we can do with this impaired balance and somebody who’s had Parkinson’s for a long time, so I think it’s really important to start thinking about this and yes, we can organize […] mediated help at home.*

Similarly, PwP shared their expectations with respect to having access to resources within the community that can support and guide them in their journey with PD. Gaining access to community resources was once again left in the hands of PwP where they had to reach out on their own and find the appropriate support in their community.

*P1: So, the example that I used beforehand was contacting the local [name of resource centre] to get a caseworker involved and to do a home assessment to assess the needs in the home and provide access to local resources, have an OT[Occupational Therapy] assessment come in, put things in, you know, like grab bars, assess the needs in the home, prevent falls, link them to local resources in [name of rural area] since they live an hour away from [name of urban centre], so we’re not quite as aware of them. That was the example I used for the life event with the [name of resource centre].*

As mentioned previously, participants mentioned the need to have resources for CP as well. This aspect was raised by CP, PwP and HCP, who recognized the importance of ensuring adequate access to supports for CP. Due to their role being viewed as critical, all participants agreed that their well-being was also important.

*HCP1: So, community resources would be, having some home care coming in, using respite if needed so you don’t have that caregiver fatigue for his wife, as they’re elderly. And then what was the other part, sorry I can’t see the full– so the respite, the home care, any extra means that they might need in [name of rural area] like the [name of food delivery service] and sometimes the [name of resource centre] is more aware of those local things than we are in [name of urban centre].*

In many instances, patients explained that when symptoms progressed and became more present in their daily life, they felt little support and faced several barriers, including financial, structural and organizational barriers.

Financial barriers specifically include the cost of therapies, medications and supplies, as well as the rising cost of these elements as part of a patient’s PD progression. In Canada, even though health care is provided through a publicly funded health care system, some specialized and privately managed services are not covered and would entail out-of-pocket expenses if patients do not have a complementary health care plan. This was often a source of stress and anxiety for many participants.

*P3: low-cost physiotherapists, especially, you know, they charge a higher rate than normal therapy which is, fair enough, with a specialist. But it’s certainly a barrier for some. […] Well I think I put down domestic support as a segue into issues of affordability for a lot of those supports.*

*HCP1: They contact the [name of health care service] to get some supports in the home—[name of health care service] is limited in what they can provide due to budget cuts and can’t offer the home health support they would benefit from—The cost of equipment in the home is high and they may not be able to afford all that is suggested to them to improve QoL and prevent falls.*

Barriers to accessing transportation services were also expressed. As mentioned previously, this challenge is amplified for PwP living in more rural or remote locations. In some cases, participants even anticipated the challenges that would arise with transportation services as they progressed in the PD journey.

*P1: At this moment, I do not have problems with transportation, like, I still drive. I am steady enough for that … but eventually that’s not gonna work.*

It was noted that during these tiring periods when symptoms became stronger, social workers were mentioned for their important facilitator role in helping and supporting PwP in their care.

*P3: I would love to have a social worker be at hand that can help guide them in the right direction. P5 remembers the period where we had a social worker in the clinic that was just a godsend [with] Huntington’s people or difficult cases. We would pick up the phone and they would, an hour later, they’d be connected with our patient, it was just awesome. It should be the way it should be so that we don’t have to do the work, but we can direct people and hook them up with social workers or other facilitators.*

#### 3.1.6. Hospitalization Episodes and Rehabilitation Time

It is not uncommon for PwP to require additional support via hospitalization. In fact, some participants, including CP, discussed the value in receiving hospital care when needed.

*CP6: That’s the thing, if we hadn’t been at the hospital, we would not have had all of those. It’s just because that’s the hospital that organized it, because they didn’t want him to go home but they wanted him to go to a special place for rehabilitation. Interviewer: Ah, okay, I understand. […] So, that’s why they organized it. I don’t think it would have been organized for just Parkinson’s, but I don’t know. I’m not sure, maybe yes, maybe no, I don’t know, but they were very helpful.*

#### 3.1.7. Journey with PD

The visual representation of the journey map allows us to see that several networks of actors (touchpoints) are identified by the participants during the care pathway of PwP (events) and these networks evolve over time by enrolling various actors (Figure 2). The journey map carried out with patients, CP and HCP, allows us to (a) identify the key actors who are at the centre of the network and how their enrolment in the network evolves over the course of the care pathway and (b) identify the barriers and facilitators that are shared by the participants in Activity 1.

The existing care network is unresponsive, fragmented, and it is difficult for PwP to find the resources necessary to maintain their quality of life. For Canada, participants draw our attention to the accessibility of services in the community and the coordination of care. For example, all the patients express the need to identify available resources specialized in PD in their community. On the other hand, some patients expressed the fact that having access to mediators greatly facilitated their journey and helped them identify resources in their community such as physiotherapists and occupational therapists.

#### 3.1.8. Various Distributed Local Networks Enrolled over Time

Some local networks (i.e., personal network, care network, community network) are constituted over time to support PwP over their care pathway. For example, at the time of diagnosis, the personal network and care network are identified as key touchpoints by the participants, but later the community network is important. We can see that the experience of PwP is rather negative during three events: at the time of diagnosis, when the symptoms and the disease progress and when PwP need more support at home and when they need to buy equipment or to arrange their environment. These are three events in the lives of patients where access to medical and social services is considered more problematic: (1) diagnostic process, (2) ON/OFF episodes and communication challenges and (3) stronger symptoms, home care and medical equipment. All patients wish to be guided, oriented and would like to have systematic access to a person contact or a single point of contact who will help them when their disease evolves. Another important element highlighted by the participants is the role played by patient organizations in Canada. Patient associations are very active and offer various resources for patients and CP by organizing webinars, conferences or offering support groups. However, the analysis of the journey map shows us that the current network is very medically oriented, forgetting the social needs of patients.

#### 3.1.9. A Medically Oriented Care Network

In its current form, the current care network is highly focused on medical onboarding of PwP. As such, there is a strong focus on immediate medical needs such as symptom treatment and medication. Still, a broader holistic onboarding is needed to account for the impacts of PD on all spheres of daily life. Participants mentioned the desire for medical-oriented care, but also support in other aspects of life which are impacted by PD.

*P3: This may be a little bit of a danger focusing too much on the Parkinson’s side and I mean there’s lots of people who need home care or some kind of home care for, you know for the whole Alzheimer’s issue for example. I mean there are worse things supposed to happen with Parkinson’s. So I guess you want to separate out what you want the specialized for the Parkinson’s and what’s much more generic should be existing.*

The organization of community services varies considerably, and it is hard for patients and caregivers to navigate through this “web of care.” PwP try to connect all the actors, services involved in the “web of care” due to the lack of care coordination or clear community linkages. This work done by patients and their families reduces their ability to manage their social and medical needs effectively, maintain independence and QoL [3]. The analysis of Activity 1 shows us is that the current care network is fragmented and distributed over several networks of actors who are not always connected to each other or who do not communicate easily.

### 3.2. Activity 2: Envisioning Integrated Care Delivery

The scenario created by the participants during the workshops present how PwP imagine an integrated care network based on at home and community models of care delivery. Based on the scenario created by the participants and the thematic analysis of the discussions, we have identified key components for designing at home and community model of care delivery in Canada. We guided our analysis using the key concepts of ANT (actors, networks, intermediaries, etc.) and our objective was to identify the key components of the integrated care network (Table 4).

#### 3.2.1. Networks Interconnected (Personal, Care and Community Network) and Key Actors Such as Family Doctors, Pharmacists, Patients’ Organizations

**Figure 3:**
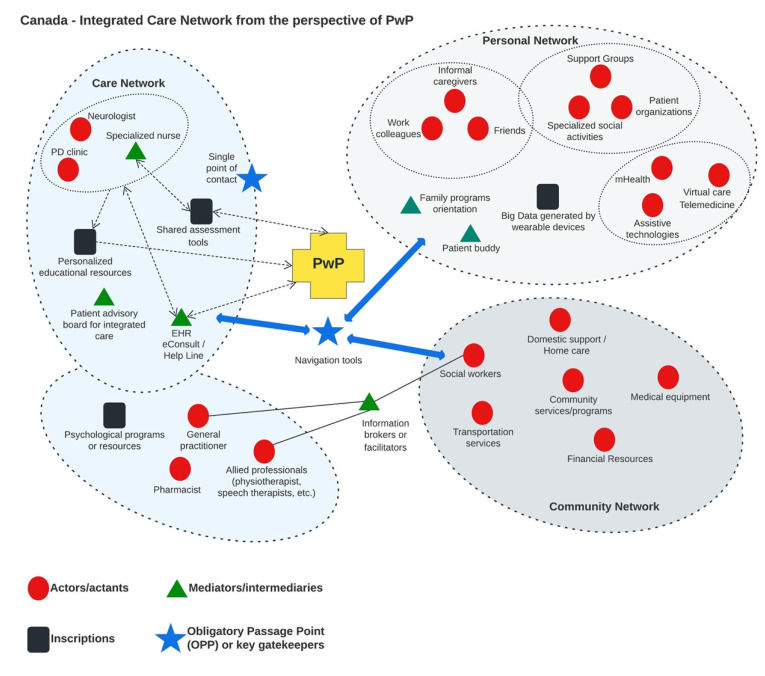
Configuration of the Integrated Care Network in Canada.

There are three main networks that must be connected to each other: personal network, care network and community network. Within each network, there are key actors or actants that could play fundamental roles such as improving communication, offering emotional support, providing tailored and personalized advice or playing the role of mediators (Figure 3).

The personal network includes not only family members but also specialized or support groups. For PwP, the purpose is not just about meeting PwP’s medical needs but about staying active and finding specialized resources within their network to maintain social activities. A lot of PwP consider the importance of joining a support group in the local patient association to create a network around them. PwP would like to use this network to discuss ideas with people in a similar situation or to share social activities.

*P3: What would be attractive to me would be some combination of support group and activity. I never became involved, but I’ve got a friend that does, in [name of city] there was some group and they do boxing and dancing.*

*P2: Dancing yes.*

*P3: It’s mainly people with Parkinson’s but not just people with Parkinson’s so. It’s just not a support group where people sit in a circle and talk about their problems [CP3, P2 and P1 chuckle]. It brings people together in an activity and then people are informally going to say “Geez my medication isn’t working well this week” or something else.*

*P2: Yeah. My family doctor has recommended that I go to that dance group.*

Family doctors or pharmacists could play an important role in the future integrated care network, especially for people living in rural or remote locations. For example, the family doctor could be a facilitator and, for some patients, may also be a resource to help them receive specialized and personalized care such as access to massage therapy, or psychological follow-up. On the other hand, for geriatric patients, this role will be more and more important when the disease evolves and the need for home care increases.

*CP3: I think one of our main struggles is how, you know, we can, be appealing for general practitioners to care for people with Parkinson’s. That’s a barrier, but in a sense, a potential could be a facilitator. But definitely, also a challenge because GPs do see– actually I think we have that feedback already with the [iCARE-PD project] that GPs see a whole gamut of patients and PD alerts are our main focus of practice… it’s a grain of sand in their ocean of patients [chuckles]. But definitely, I think both P5 and P1, you know, we end up seeing ourselves being the GPs of our patients, which also shouldn’t happen, so how you strike this balance and make family doctors more at ease of managing problems that do happen let’s say in the geriatric population, but not necessarily specific of Parkinson’s, and so, in a way, but they could also manage those problems for example.*

*CP5: So, I guess maybe one of the keys, as P3 was saying, not every patient was saying, not every patient needs all of these services, but I do think if you look at our red boxes, every patient with Parkinson’s, I feel an essential need is a general practitioner. For many patients in smaller communities that’s still unfortunately easier said than done. But I think that’s critical. This is not a condition that is going to get better and is going to get worse and we know all the non-motor things, definitely having the family doctor involved early and feeling more comfortable and knowing the patient can really make a big difference to how the patients are doing, because of, you know, treating their depression and really having a really strong family doctor can be a real key. And again, I think with our Canadian health care system is something we need to make sure is a real part of this process.*

Patients believe that some technologies (such as mobile applications, telehealth or assistive technologies) will play an increasingly important role in the future and will be key actors in the personal network. These technologies will provide access to online resources (e.g., online physiotherapy programs or online exercises), but also serve as a communication tool with the care team to maintain proximal communication. Moreover, access to these technologies can allow people living in rural areas to benefit from online services and thus reduce certain inequalities of access that some people living far from large urban centres may encounter.

*P4: so, for me virtual reality has opened up access to expertise and, you know, having resources that you wouldn’t typically have, for me that would be a big bonus. Although saying that I live in [name of rural area] and can’t do virtual reality from my house since we have terrible Wi-Fi reception. So, it opens up in that avenue, but if you have terrible Wi-Fi, then there’s a limitation and a barrier to that.*

*P5: how am I ever going to know who’s the physiotherapist that’s within five kilometres of our patient who lives in a small town somewhere in Canada? There’s just zero chance I know that and then who is going to help us figure that out? And I think this is where technology is the only solution. Even in [name of city] we can’t keep a running database of all the physiotherapists, let alone the social workers, let alone the occupational therapists, so I think we have to engage technology to help us come up with solutions for this because for 15–20 years I’ve tried to keep a list of these types of things and it doesn’t work.*

#### 3.2.2. OPP: Single Point of Contact and Navigation Tools

Two components are identified as OPP, i.e., as central actors that will contribute to the creation of the integrated care network, its personalization and maintenance over time. So, these are critical network channels and fundamental components of an integrated care network. A single point of contact (i.e., specialized nurse), a person connected to the neurology clinic, is an essential element to bring different actors into the network in order to personalize the medical and social care offered to patients.

*P7: Having the ability to contact someone to get some information if I’m not able to find it myself.*

*HCP3: I think a wonderful model would be if there’s a nurse coordinator who interacts with the patient, and she identifies four things that should be done: education, physiotherapy, maybe day programs or whatever. So, she fills out four forms for pharmacy, optimization of drugs.*

Another key element of the network is a “resource browser” (navigation tool) which could take different forms such as “iCARE resource finder” as suggested by one participant. This is a tool that could be shared and accessible to both health care professionals involved in the network and PwP.

*HCP2: [is] key you know, building this network of resources by having ways to obtain that information, in a certain geographical area, but I would say [name of rural area] would be one, and it’s not that far from [urban centre] and we receive patients from that area. And of course, the challenge is not only how we identify them, but actually maintain that tie-in. So iCARE finder resource that allow us to help these patients navigate whatever resources are available. […] And so, I guess that’s one potential facilitator, but it’s definitely challenging but then, the likelihood of having resources in [rural area] I think it’s less, at least, not in terms of the sheer number but also that the degree of expertise in Parkinson’s disease, for example.*

#### 3.2.3. Key Intermediaries or Mediators: Specialized Nurse, Advisory Board, Information Brokers, Programs Orientation, Specific eHealth Technologies Improving Communication with HCP

The future integrated care model must be a model of care that relies on a close relationship with a specialized nurse because interpersonal communication is central for PwP. This is the basis of a humane model of care and allows for personalized management throughout the course of the disease. It is therefore essential to pay attention to the role played by the nurse as an important intermediary or mediator within the network. The specialized nurse will act as an intermediary or mediator between the different networks identified (personal, care and community networks). They are responsible for coordinating care by being a key player in the care network, developing personalized educational resources for PwP, but also for helping patients navigate the network by identifying tailored resources in the community network. To do this, specialized nurses will need to be trained and equipped to maintain close communication with patients and their families.

*P2: it might become an old-fashioned idea, but I do think patients, they really cherish the physical patient-physician relationship. A lot could change but definitely having these direct interactions I think it’s very important.*

*CP4: So for me, I would like to see the nurse coordinator as P1 is saying, to then spread out and bring out a multidisciplinary team on board depending on where that person is on their journey. […] I’ve been working for a while and a lot of times the nurse coordinator was a team coordinator that would link up with the other professionals in a said team, and whatever that team is. Right, so that’s looking probably at health care in the past, where you had a team, part of a movement disorder clinic. Whether that’s in-person or virtual.*

*P2: I think a lot of it has already been said, but perhaps I like the idea of the curation of how can we help the patient navigate in the wealth of information and I do agree that information is key but it can also be toxic in a way. And so, what’s the role of we health care professionals in helping to navigate that wealth of information?*

To keep the integrated care network adapted to the needs of the patients and their families, PwP propose an organizational structure involving a patient advisory board that would allow an evaluation and adaptation of the network over the years. This could aid in assessing and improving its effectiveness.

*P2: I think the idea, we had that idea when we created the advisory board for the [name of network], but, of course, that could be expanded in almost like a forum or, you know, create times where you can actually brainstorm with patients and CP of how they seek care, right? And so, what P3 is saying, expanding this idea of a patient advocate or a patient advisory board, right, and, but be part of, on a regular, I guess, be part of the structure of the care delivery model, right, where there’s a more regular interaction with patients and CP.*

Patients draw our attention to the importance of not centralizing the network in large urban centres but adapting it to people living in rural areas who may not have access to transportation services. Thus, patients suggest identifying “facilitators” or “information brokers” within the community. This role could be assumed by a social worker who is familiar with community resources and who could be a point of contact for patients and their families.

*P3: Depending on, well ideally, I think it would be nice to have the person in the clinic, but it doesn’t have to be. If I had someone in [name rural area], who I can call because I know he or she is a social worker who’s doing a great job with the community members, he or she does not have to be at the [name of facilities in urban centre]. […] I don’t think the social worker has to be involved for every person, but we should have access to that menu option as well is what I’m trying to say. […] it’s very much like a drop-down menu where you pick what is the best match for what the person needs in the near future.*

An important resource within the integrated care network is the creation of tailored programs for orientation (i.e., family orientation programs) that address specific needs of PwP. For example, patients suggest that a program could be developed to support patients and CP through the various patient trajectories. For example, a program could be tailored and personalized for newly diagnosed patients to help them understand their disease and address their specific needs, as well as for patients whose disease is progressing and who need to anticipate their future needs such as home care. These programs are not only for individual patients, but also for their caregivers. It is therefore a collaborative approach that is favoured by the patients, PD is a disease that has an impact on family and social life. Therefore, offering and designing specialized programs are important components of the integrated care network. Indeed, integration also involves the family and a better integration of the personal network of people living with the disease.

*CP3: And whether it’s a spouse or it’s another care, somebody who is stepping up to play a role, like a child, an adult child or. Yes, obviously I think our, the support, support system or ideal care system should include, you know, a family orientation. If a family, if that’s what the, you know, the individuals choose.*

For patients, specific eHealth technologies will have a role to play in the future and will be important intermediaries to facilitate the coordination of care, but also communication with the care team. Unlike the technologies mentioned as key actors within the personal network, these technologies mentioned here will play a very specific role in facilitating communication and care coordination. They will therefore play a more important intermediary and mediation role within the care network. The possibility of having a help line or online consultations is an important component of the network in the future. However, the current network needs to restructure itself to be able to offer this service, which also requires human resources and infrastructure.

*P3: I just find telephone communication is not effective. There has to be another way and I think virtual interactions, which is then difficult to organize because there has to be a booking, there has to be a Zoom link, you have to be online, and that in itself take a lot of time of admin people or doctors or nurses. I have absolutely no idea how to solve this. The better we are in helping people with an integrated care approach, it’s so time and resource consuming. I don’t have anything productive to add is what I’m trying to say.*

*P1: So, in terms of technology, I would say that telemedicine has been really good at having easier access to the physicians to get advice and just those virtual appointments have been really nice especially for those, was trouble with mobility or access to rides to get to appointments and stuff. I think virtual appointments have been really good for patients.*

*P2: I find that now with my chart at the [city] hospital anyway, you can go in and look at your test results and things like that. If you understand what it means. So I will go in there and look at reports and some things I had to fill out for various doctors. I also have the telehealth and mobile applications so I just find any technology, the more technology you can have, you know what it means, it’s useful to have, at times.*

#### 3.2.4. Inscriptions

The creation and use of various forms of inscriptions (i.e., assessment tools, tailored educational resources, data generated by eHealth devices) by the integrated care network contribute to better defining care pathways in personalized ways. Shared assessment tools that will allow the care team to define with the patient and his family a personalized care pathway and support an informed decision will have to be created and shared.

*HCP3: the integration of the nurses and the physicians’ assessment which services should be utilized, accessed, and at that time put in place. And so, you know, I think because this so much depends on the individual and on the family, right. So, you don’t have one model that fits everybody, but you have the option of a whole list and menu, and you pick and the nurse’s perspective and the doctor’s perspective, and the patient and his family’s perspective cross off these overlapping needs, or the needs that have been collectively identified, that would be the best-case scenario for me to access the resources available.*

At the heart of the future integrated care network are tailored educational resources that address the needs of different patients’ trajectories. Trajectories that we have identified in the early phases of the co-design process [22]. These educational resources will play an essential role in supporting the self-care process throughout the patient journey.

*P4: Education for me is a huge thing and one of the big areas of focus that I would like to see is more along the lines of chronic disease self-management programs that Stanford have piloted, done with educational workshops for those, in major diseases, but certainly some aspect of understanding chronic disease self-management program and whether that’s through, as P5 and P2 said, the [name of the organization] or through [name of the organization] or linking up with experts from your movement disorder clinic but for me that would be a big role that I would like to see.*

One element to consider in the development of an integrated care network is the role that eHealth technologies may play in the future. For example, some technologies such as sensors or wearable devices that collect data in real time and generate big data, will contribute to improving the personalization of medical care (e.g., personalized medicine based on big data).

*P2: I think the wearables, although they have a lot of potential, we’re still trying to understand exactly what we use it for even just measuring Parkinson’s disease and its different dimensions. And then, of course, the next step, how can we use it for care, so I think that will really take longer, it will be a longer process but is still valuable, right? If ideally, we could, technology could help us to understand what’s happening to people living with Parkinson’s between, you know, clinical visits, that will be huge, but I again, I don’t think you’re interested in getting the big data, 20 pages of a report, but in an eloquent, synthetic way can technology gives us a portrait of how, a snapshot of how the patient has been doing in the six months prior to seeing them in person.*

The ANT analysis allowed us to identify key components within the network of care designed by the participants in Canada (Figure 3). The integrated care network designed by the participants is composed of three interconnected networks, where key components play an important role to support personalized care in PD. The integrated care network co-designed by the participants must be constituted, made and remade over time to evolve with the patient trajectories (Figure S5 [22]).

## 4. Discussion

As mentioned previously, the analysis of Activity 1 illustrates a current care network that is fragmented and distributed over several networks. This main problem was recognized by PwP who shared their experiences in navigating a system composed of mediators and intermediaries as actors who are not always connected to each other or who do not communicate easily. Such a research project is valuable in addressing gaps and proposing solutions to lead to an integrated care network that is truly based on patients’ needs.

To optimize and personalize the care for PwP, we need an integrated care network that is tailored to patients’ needs, with three core components: (1) OPP, (2) mediators or intermediaries and (3) inscriptions. We discuss each of the three core components, followed by a discussion on their relevance and practical implications to support personalized care for PD.

### 4.1. OPP as “Boundary Spanners” to Enroll Actors

OPP refers to an important point of contact that channels all interests into one direction [9]. The first stage of the constitution of the network around the patient involves key actors (i.e., single point of contact and navigation tools) identifying the needs and interests of PwP. This actor could be human or non-human and becomes the OPP, making themselves indispensable within the integrated care network.

In our study, specialized nurses are the focal actors in the integrated care network. As part of their work, assessment tools (intermediary) can be used to identify patients’ needs and build a partnership with the patient, their family and other actors in the community. Their role therefore promotes shared decision-making and contribute to personalizing the care pathway. Our findings align with recent literature on the role of specialized nurses as facilitators with expert skills within integrated care networks [2], [32]. A single point of contact and navigation tools are indispensable for the constitution of an integrated care network and the stabilization of the network over time by supporting a “system of navigation and alliances” inside the network [8]. The role-playing by the OPP is fundamental for exploring how other actors within the network are enrolled progressively.

### 4.2. Intermediaries to Connect Actors in the Networks

At the heart of the network, there are intermediaries or mediators who will play an important role in promoting communication and information exchange. There are four important intermediaries: specialized nurse, advisory board, programs orientation and specific eHealth technologies for improving communication between care team and patient (e.g., electronic health records such as EHR, and online support via eConsult, or Help lines). These technologies will improve communication with the care team and facilitate the exchange of information. They will therefore play a very specific role and will be part of an informational and technological infrastructure that must be put in place to allow for more patient-centred care.

Within the personal network, two intermediaries are identified as being able to play an important role in the constitution of a personal network that will play an important support role in daily life. First, the implementation of a program designed for the family (e.g., Family Orientation Program) at the time of diagnosis and as the disease progresses could allow the patient and his or her caregivers to find both emotional support and information resources that will allow them to better understand the disease and anticipate future needs. This aligns with previous research that emphasizes the importance of supporting the family structure, which is especially helpful for CP in that it aids in reducing caregiver strain [33]. Other research also reflects on the importance of providing caregiver assistance and support [34,35], which aligns with our findings that indicate the need for family-oriented approaches within the community. The envisioned program could be designed and organized in collaboration with the neurology clinic and Parkinson Canada, which is a patient organization well established in Canada and offering services in both official languages. This program could offer seminars for newly diagnosed patients and their families or specific information sessions for families as the disease progresses and new social and medical care needs emerge. The objective of this program is to offer highly targeted resources at key moments in the patient’s trajectory, i.e., at the time of diagnosis and when symptoms progress, and PwP’s need to be better informed and accompanied to find resources within the community network.

Another important intermediary that connects networks is what we call “information brokers.” These facilitators can be different actors within the network depending on the needs of the patient (e.g., rural or urban location, trajectory with the disease, services offered in the community). For those living in rural and remote locations, several options have been described in the literature as viable and facilitating access to care and health information [36]. For example, advances in virtual care through telemedicine or nurse-led clinics are opportunities that allow patients and families to receive support even when they live in more rural and remote locations [36]. For some patients who have a good relationship with their general practitioner, they could take on this information broker role. Recent literature describes the role of general practitioners as pivotal in the care for PD [37]. These HCP have access to various tools, guidelines, and benefit from proximity with patients which allows them to make recommendations and suggestions that can improve the well-being and quality of life of patients and their families throughout their patients’ journey with PD [37]. For other patients who have a more complex disease trajectory and require more resources in the community, a social worker could be that facilitator. It will therefore be up to the care team to identify, with the patient and their CP, the actor within the community network who can assume the role of facilitator or information broker. This could be the general practitioner, the social worker or other allied health professionals.

### 4.3. Inscriptions to Support Tailored Care Network and Improve Communication Process

Inscriptions is a process in which the translation of actor interests is embodied in artefacts such as texts, technical objects, embodied skills, and management tools [9]. Any component of the heterogeneous network could support the constitution of a social order and may be the material for inscriptions. The stability and social order, according to ANT, are continually negotiated as a social process of aligning interests. Inscriptions represent interests inscribed into an artefact or a tool. As Law [8] points out, “thus a good ordering strategy is to embody a set of relations in durable materials. Consequently, a relatively stable network is one embodied in and performed by a range of durable materials” (p. 387).

As identified in Figure 2, several inscriptions were identified by participants as key components of the integrated care network. The actors present in the different networks may have diverse interests (e.g., patients want to be able to finance improvements to their homes and adapt their environment, and the medical team wants to monitor the evolution of the disease and define the best possible treatment). However, the stability of the network depends essentially on the ability to translate, i.e., to reinterpret, the interests of others into one’s own. This work of translating the interests of others presupposes tools, programs such as assessment tools, programs or resources that will support this process of translating interests and needs. This will then be embodied in texts, tools, which become the support of this work accomplished by the actors together to create and maintain the network. These inscriptions co-produced and shared are also communication tools that will circulate within the network and support the coordination work.

### 4.4. Practical Implications

**Figure 4:**
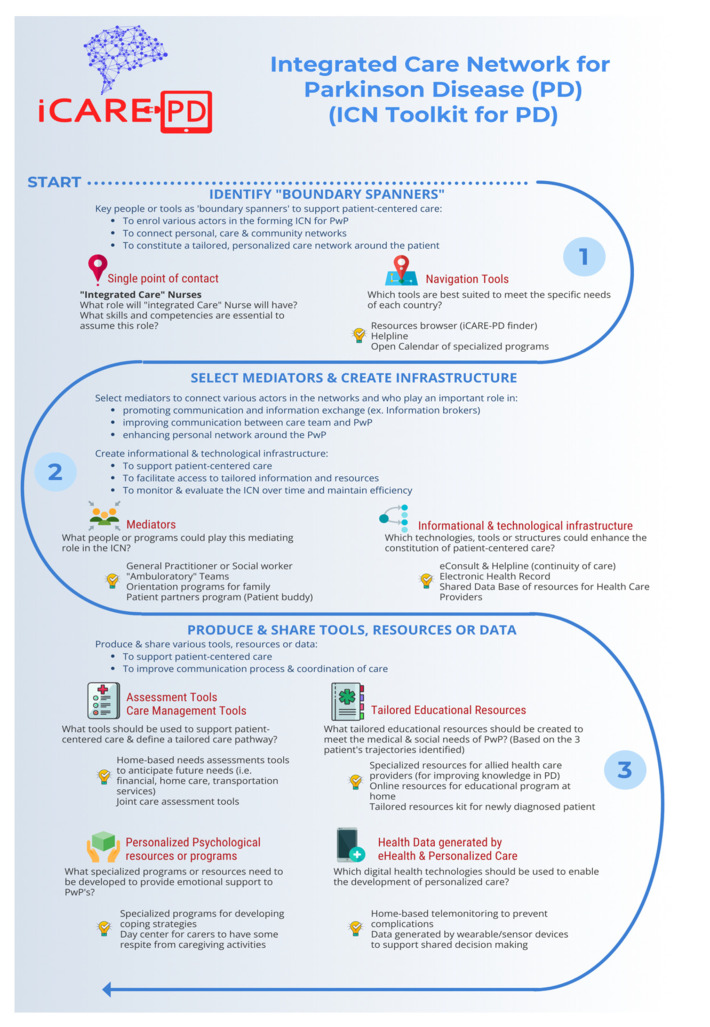
Roadmap and toolkit for the constitution of the iCARE-PD project in Canada.

In support of recent literature on the value of outpatient integrated models of care [5], our study helps delineating key components of integrated care networks. For each component, we have identified management tools, skills or educational resources that could be created or used in the future by this integrated care network. Thus, the co-design workshops allowed us to generate concrete and practical solutions that will be discussed in the final co-design phase with the different stakeholders of the iCARE-PD project. To improve person centredness, tailor and personalize care and propose a holistic approach of PD in integrated care, three design features have become relevant: (a) identify ‘boundary spanners,’ (b) select mediators and create informational and technological infrastructure and, (c) produce and share tools, data and resources. Each of the design features involves various practical solutions that could be useful to develop and implement an integrated care network that considers the three main patient trajectories identified during the initial phase of the co-design approach. The practical solutions suggested are drawn either from the scientific literature or from suggestions, ideas made by the participants during the co-design workshops. We discuss a few examples suggested in Figure 4.

#### 4.4.1. Examples of “Boundary Spanners”

Single point of contact: It could be a service centre that provides a single-entry point to access specialized care team. For Canada, the service centre involves a specialized and trained nurses coordinator who will play the crucial role of “boundary spanners” [38].

Navigation tools: These tools could take many forms and be adapted to the local and geographical reality. A key aspect of supported self-care includes the enabling of PwP to connect with ongoing personal and community networks. As part of the iCARE-PD project in Canada, one suggestion could be to develop various tools to support navigation. One suggestion proposed by the participants is the iCARE-PD finder resource, and a shared tools that support care navigation and linkage with community services.

#### 4.4.2. Examples of Informational and Technological Infrastructure and Mediators

Family orientation programs: The idea is to create an informational infrastructure based on key tailored programs that can both inform and guide PwP and connect them to their various networks. Considering the family orientation is critical and has been emphasized in the literature, especially in the benefits it may provide for CP.

Advocacy Board: A continuous monitoring and evaluation of the integrated care network is required to maintain the efficiency of the model of care. Patient advisors, community representatives and other stakeholders will be included as members of the board. Consultations with people living with PD and community representatives are an opportunity to engage various actors and discuss new challenges faced by PwP and their families.

eHealth technologies: Teleconsultation, tele-monitoring or telerehabilitation are helpful tools for creating an integrated care infrastructure and positively increasing patient satisfaction and improving continuity of care and communication with the care team. The role of these technologies is also described in the literature as important care delivery models to connect individuals from rural and remote locations [36], and in exceptional circumstances such as the COVID-19 pandemic [39].

#### 4.4.3. Examples of Tools, Data and Resources

Shared assessment tools: Some joint care assessment [40] or shared care planning assessments exist to explore the health and social care needs by involving patients and their CP. Based on the result of this assessment, a draft care plan could be co-defined and adjusted based on the PwP personal goals, needs and expectations. This personalization allows us to identify unique personality traits and coping styles that impact care in PD [41]. However, the implementation of this tool requires trained and specialized nurses who are able to assess medical and social care needs. It is also possible to imagine the development of a digital tool that can be shared between the care team to better coordinate care.

### 4.5. Limitations and Future Research

The roadmap and toolkit proposed in this article is based on the results of a Canadian case study. As such, it represents the views and experiences of PwP, CP and HCP living in Canada specifically. The roadmap could be supplemented by incorporating the results of the multinational co-design approach implemented in Germany, Czech Republic, Ireland and France. The roadmap and toolkit created serve as a preliminary guide for planning and implementing an integrated care network for people with PD (iCARE-PD) and outline key design features or components that support tailored and personalized care. The social, cultural, geographical and political context of the implementation of an integrated care network must be considered and the network must adapt to these local realities. For this reason, the elaboration of a roadmap and toolkit does not allow us to define a static and fixed model, but to propose key elements that must compose the network to make it adaptable and flexible in different contexts. Future research could focus on the validation, adaptation, implementation, and evaluation of the roadmap and toolkits.

## 5. Conclusions

The analysis of current experiences in the journey with PD and co-designing a vision for the future of care has shed light on key components that should compose an integrated care network. OPP in the form of boundary spanners such as specialized nurses, intermediaries in the form of mediators and the creation of infrastructure such as technological or informational infrastructure, and inscriptions in the form of health data, resources or tools such as tailored educational resources are critical. In Canada, this means that redefining the role of a specialized PD nurse, where clear sets of roles and competencies are highlighted, creating more personalized care by using health data from technologies such as wearables, and developing educational resources that are interactive and allow searching for community supports, represent tangible opportunities to advance work toward an integrated care network for PwP.

Our results also highlight the need to consider the future development of therapeutic education tools and support programs for both patients and their care partners or families. Thus, it is relevant to pursue, especially in Canada, a close collaboration with patient associations to propose tools and support programs that are adapted to the different profiles of patients (living in urban or rural areas). The current challenge for the integrated care network is to be able to adapt and offer tools and resources that are adapted to the needs of remote communities and people with a lower socio-economic status.

Another element that our study highlights is the importance that the constitution of technological and informational infrastructure will have in the future to support the development of personalized medicine (personalization of treatments based on health data generated by technologies such as sensor devices or others), but also close communication with the care team to make the network less reactive and more proactive.

## Figures and Tables

**Table 1:** Sample characteristics, *n* = 15.

PwP *—Characteristics		*n* = 10
Gender	Female	5
	Men	5
Age	≤50	0
	50–60	1
	61–70	4
	≥71	5
Stage of the disease	Stage 2	5
	Stage 3	4
	Stage 4	0
	Stage 5	1
Years since diagnosis	≤2 years	1
	(2 years ≤ 8 years)	8
	>8 years	1
**HCP—Characteristics**		***n* = 5**
Physiotherapists		1
PD nurse		1
Neurologists		3

* 3 PwP chose to be accompanied by CP (*n* = 3).

**Table 2:** Summary of key concepts used in ANT.

Concepts	Definitions
Actors/actants	Human or non-human entities that interact within networks of other actors. Actors could be individual or collective.
Networks	Collection of actors that interact, form, and align with each other for the purposes of accomplishing actions or tasks.
Intermediaries/mediators	An individual or object that serves as a connection between two actors.
Translation	“Translation consists in one particular actor being able to act as the spokesperson for the many others it manages to enroll in a particular program of action.” [29] (p. 5). The process of translation includes four stages: (i) problematization (bring together actors with common interests), (ii) interessment (convince other actors to play a role in the newly emerging network); (iii) enrollment (when actors accept to play a role in the network); (iv) mobilization of allies.
Obligatory Passage Point (OPP)	A dominant actor that becomes a gatekeeper between other actors in the forming network.
Inscriptions	Inscription is the process that ascribes meaning to artefacts. Inscriptions could be material elements such as documents but also practices, rules, routines or skills.

**Table 3:** Thematic analysis of Journey Mapping (Canada).

Events	Diagnostic	Impact on Work, Trips or Activities	Treatment Plan or Change	ON/OFF Episodes or Communication Challenges	Stronger Symptoms, Home Care, and Medical Equipment	Stop Driving, Buy Medical Equipment	Hospitalization Episodes & Rehabilitation Time
**Touch** **points**	*Personal Network* Family and Friends *Care Network* General Practitioner (GP) PD Clinic PD Nurse *Tools* Booklet about PD *Technologies* Website about PD & Internet	*Personal Network* Family and Friends Parkinson Patient & PD associations *Care Network* GP Physiotherapist *Community Network* Exercise Groups Sports complexes	*Care Network* PD Clinic PD Nurse Pharmacist Allied Health Professionals (i.e., Physiotherapist Alternative medicine Professionals) *Technologies* Websites about PD & Internet	*Personal Network* CP and Family Formal caregivers *Care Network* Allied Health Professionals (i.e., physiotherapist, speech therapist) *Community Network* Community Programs & Resources centres (Volunteers) Community Parkinson’s Group—Networking Group *Technologies* Internet	*Personal Network* CP and Family *Care Network* GP Allied Health Care (i.e., Physiotherapist Occupational therapist Massage Therapist) PD Nurse *Community Network* Community Programs Care at home program Social worker Transport services *Technologies* Help Line/Phone Electronic Health Record (EHR) Virtual care	*Personal Network* CP and family Friends *Care Network* Occupational therapist Physiotherapist *Community Network* Transport services Community Programs Social worker *Technologies* Information resources (online or in accessible format)	*Care Network* PD clinic PD nurse Rehab Program Physiotherapist *Community Network* Community Programs/Exercise Group Parkinson’s Association *Technologies* EHR
**Barriers**	*Lack of support/* *guidance* -Difficulty identifying who is better suited to address specific needs or issues. -Lack of clear direction before & after diagnosis. *Lack of knowledge about PD* (family and GP). *Lack of trust* in the care team (e.g., first neurologist). *Inequality of access* -Long wait time to see a neurologist	*Lack of guidance to navigate local resources* -Lack of knowledge about local resources *Personal attitude & condition* -Stress -Concerns about bothering people—Taking up time from other people	*Lack of PD expertise in the community services*	*Personal skills or attitudes* -Safety concerns limiting access to community resources and activities -Computer literacy *Lack of guidance to navigate local resources* -Lack of knowledge about local resources *Lack of PD expertise in the community services*	*Treatment efficacy* -Medication less effective -uncontrolled symptoms -Pain, anxiety or loss of independence *Environmental constraints* -Technological difficulties (needing to do virtual consultation) *Lack of support/guidance* -Limited help—respite for informal caregivers *Inequality of access* -Waiting time to access day programs (e.g., Bruyère Day Program) -Lack of transportation services -Lack of financial resources *Lack of PD expertise in the community services*	*Inequality of access* -Lack of access to transportation services -Lack of financial support to buy medical equipment -Waiting time to access occupational therapist or care at home *Lack of support/guidance* -Limited help—Respite for informal caregivers *Environmental constraints* -Lack of mobility and risk of social isolation)	*Inequality of access* -Long wait time at local hospital (not specialized PD clinic) -Lack of support for CP and family -Distance from PD clinic *Personal condition* -Loss of autonomy and independence *Lack of PD expertise in the community services*
**Facilitators**	*Single point of contact* -Having a contact person to ask questions (nurse or general practitioner). *Easy access to information* -Easy access to information (via mediator or personal research) -Personal information-seeking behaviour	*Personal network* -Support from family & friends. -Support groups (PD associations). *Access to educational resources* -Educational resources about PD & treatments available	*Positive and trusting relationship with the care team* -Access to caring & knowledgeable health care professionals *Treatment efficiency* -Appropriate medication to control symptoms -Access to alternative medicine *Single point of contact* -Mediators to connect PwP with community services -Virtual visits (distance from clinic) *Access to financial aid*	*Access to useful & tailored educational resources or programs* -Improve patient education by focusing on tailored needs -Sufficient knowledge to get appropriate information -Online support groups *Access to caring & knowledgeable health care professionals* -Maintain close relationship *Single point of contact & information brokers*	*Access to home support* -Care from other individuals—respite care *Access to local clinic* (convenience) *Single point of contact* -Mediators to connect PwP with community services (informal caregivers) *Easy communication channel* -Emergency phone call (Help line) *Accessible environment and equipment*	*Single point of contact* -Mediators to connect PwP with community services *Access to home support* Access to home support Access to financial aid *Easy access to information* Via mediators or personal research	*Single point of contact* -Mediators to connect PwP with community services *Access to home support*

**Table 4:** The key components of the integrated care network (Canada).

Core Themes Based on ANT	Subthemes		
Networks	Personal network/Care network/Community network		
Actors/Actants	*Personal network*	*Care network*	*Community network*
	Informal caregivers	PD clinic	Community services or program
	Work colleagues	Neurologist	Domestic support or home care
	Friends	Specialized Nurse	Transportation services
	Supports groups	Allied professionals	Social workers
	Patient’s organizations	GP	Financial resources
	mHealth	Pharmacist	Medical equipment
	Assistive technologies		
	Virtual care		
OPP	Single point of contact and Navigation tools		
Intermediaries Mediators	*People*	*Organizational structure and programs*	*Technologies*
	Specialized or PD nurse	Patient buddy	Family orientation programs
	Facilitators/information brokers (i.e., social worker, GP)	Patient advisory board for iCARE-PD	HER—eConsult/Help Line
Inscriptions	Shared assessment tools Tailored educational resources Emotional/psychological programs or resources Sensors/wearable devices (big data and precision medicine)		

## Data Availability

The data presented in this study are available on request from the corresponding author. The data are not publicly available due to ethical reasons.

## Supplementary Materials

The following supporting information can be downloaded at: https://www.mdpi.com/article/10.3390/jpm12061001/s1. Figure S1—Journey Map completed by PwP; Figure S2—Inspiration Card completed by PwP; Figure S3—Example of Persona; Figure S4—Online Inspiration Card with HCP; Figure S5—Patients’ trajectories identified in [22].
