# Supplementary figures and images for: Co-Designing an Integrated Care Network with People Living with Parkinson’s Disease: A Heterogeneous Social Network of People, Resources and Technologies
Source: J Pers Med. 2022 Jun 19;12(6):1001. doi: 10.3390/jpm12061001 (PMC9225240; doi:10.3390/jpm12061001)

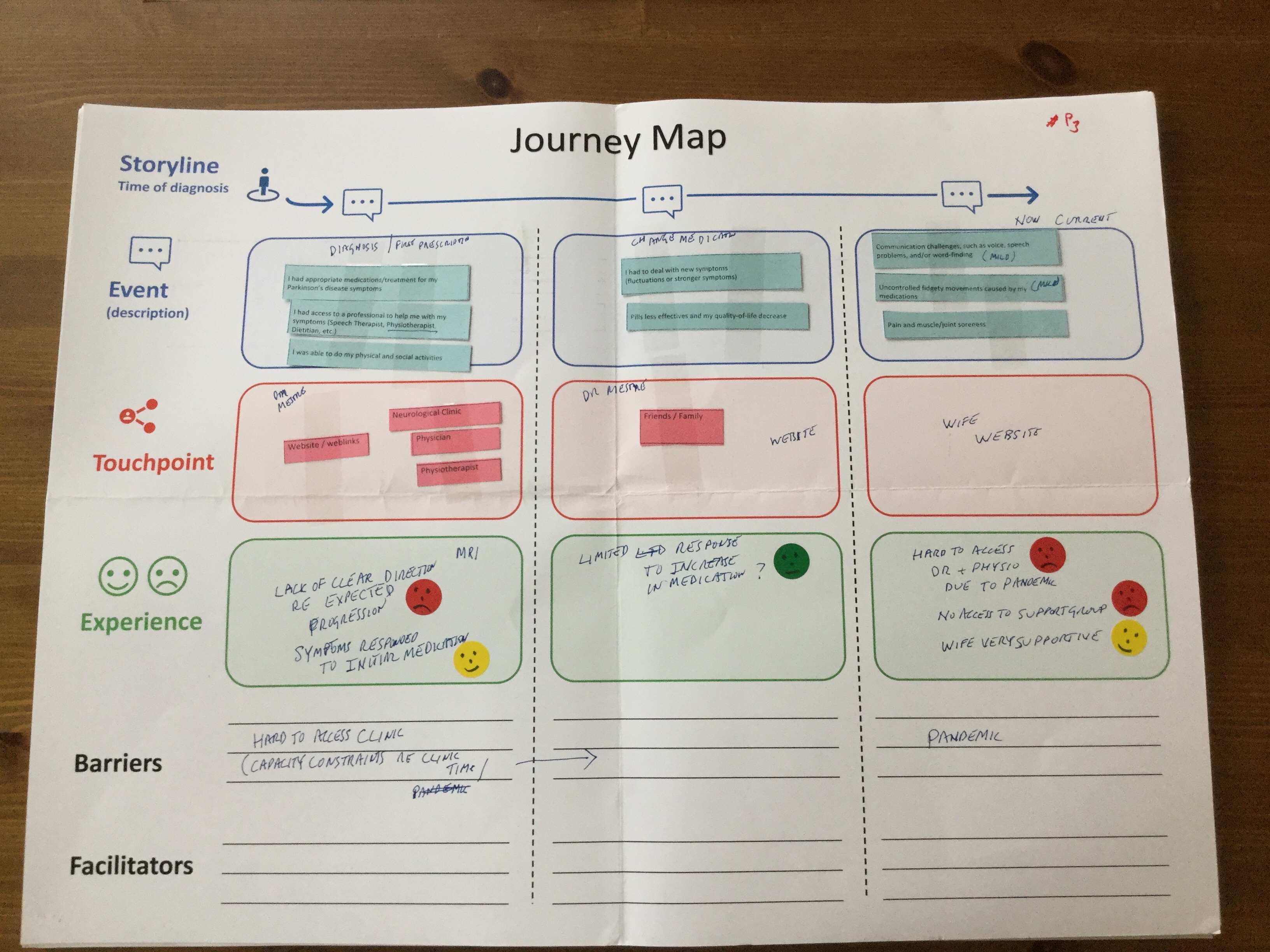

Supplement: Supplementary file 1 [file jpm-12-01001-s001.zip › Figure S1 í¬Journey Map completed by PwP.JPG]

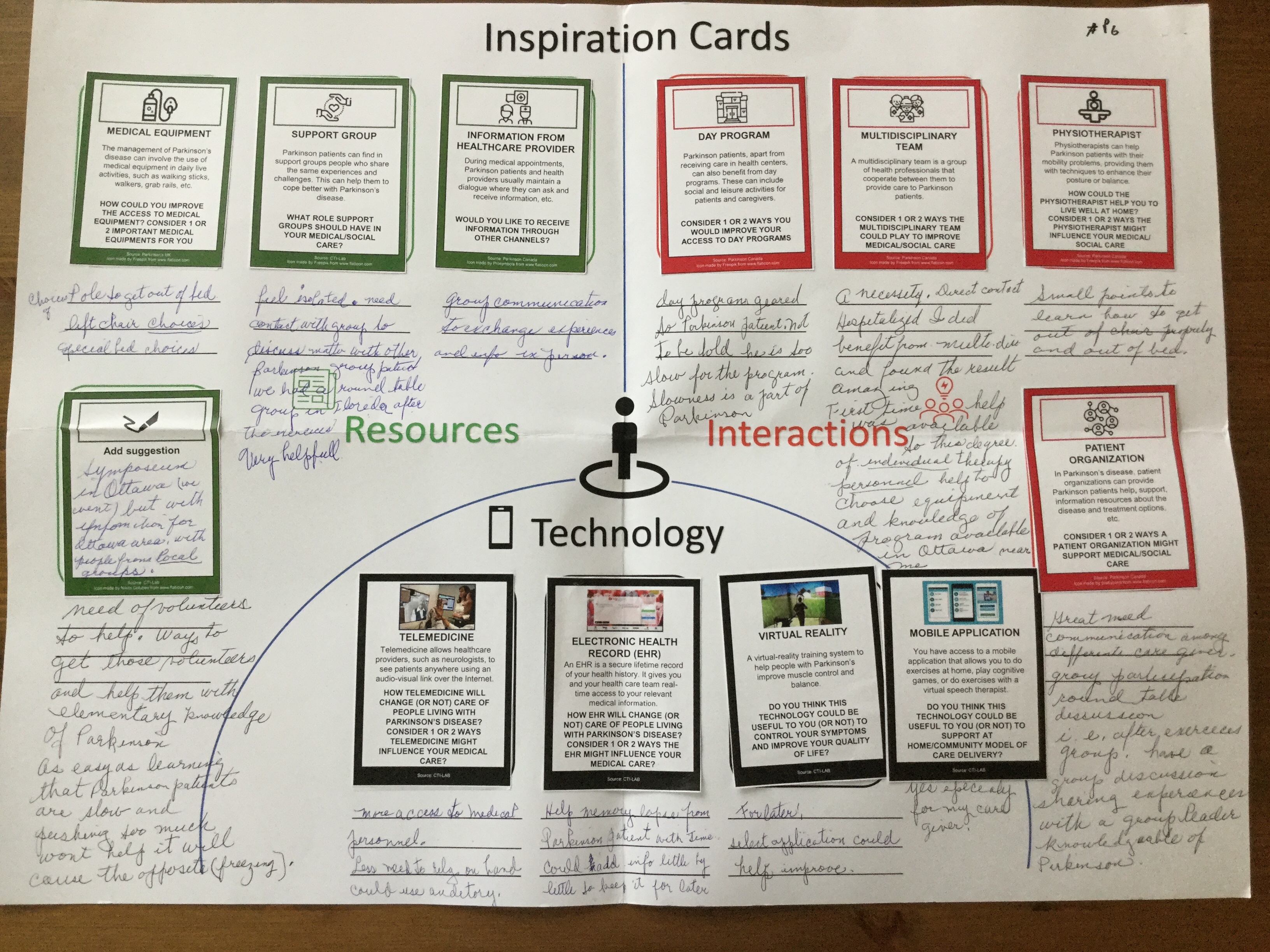

Supplement: Supplementary file 1 [file jpm-12-01001-s001.zip › Figure S2í¬Inspiration Card completed by PwP.JPG]

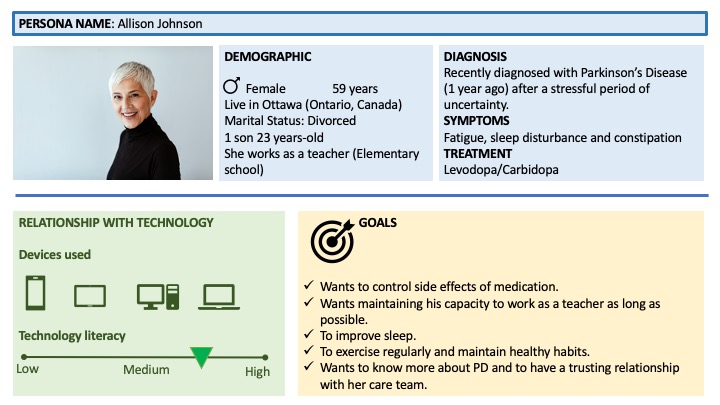

Supplement: Supplementary file 1 [file jpm-12-01001-s001.zip › Figure S3í¬Example of Persona.jpg]

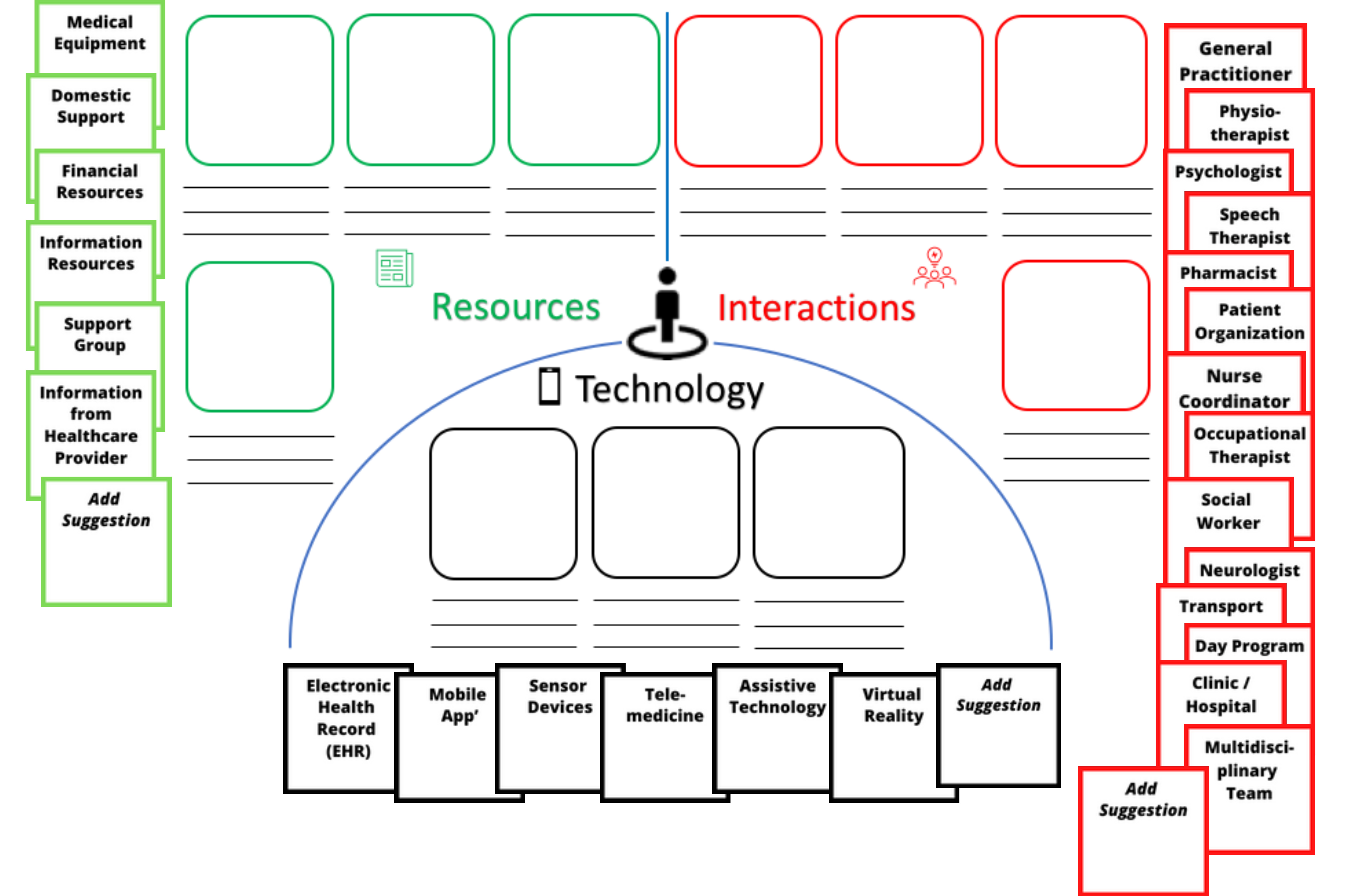

Supplement: Supplementary file 1 [file jpm-12-01001-s001.zip › Figure S4í¬Online Inspiration Card with HCP.jpg]

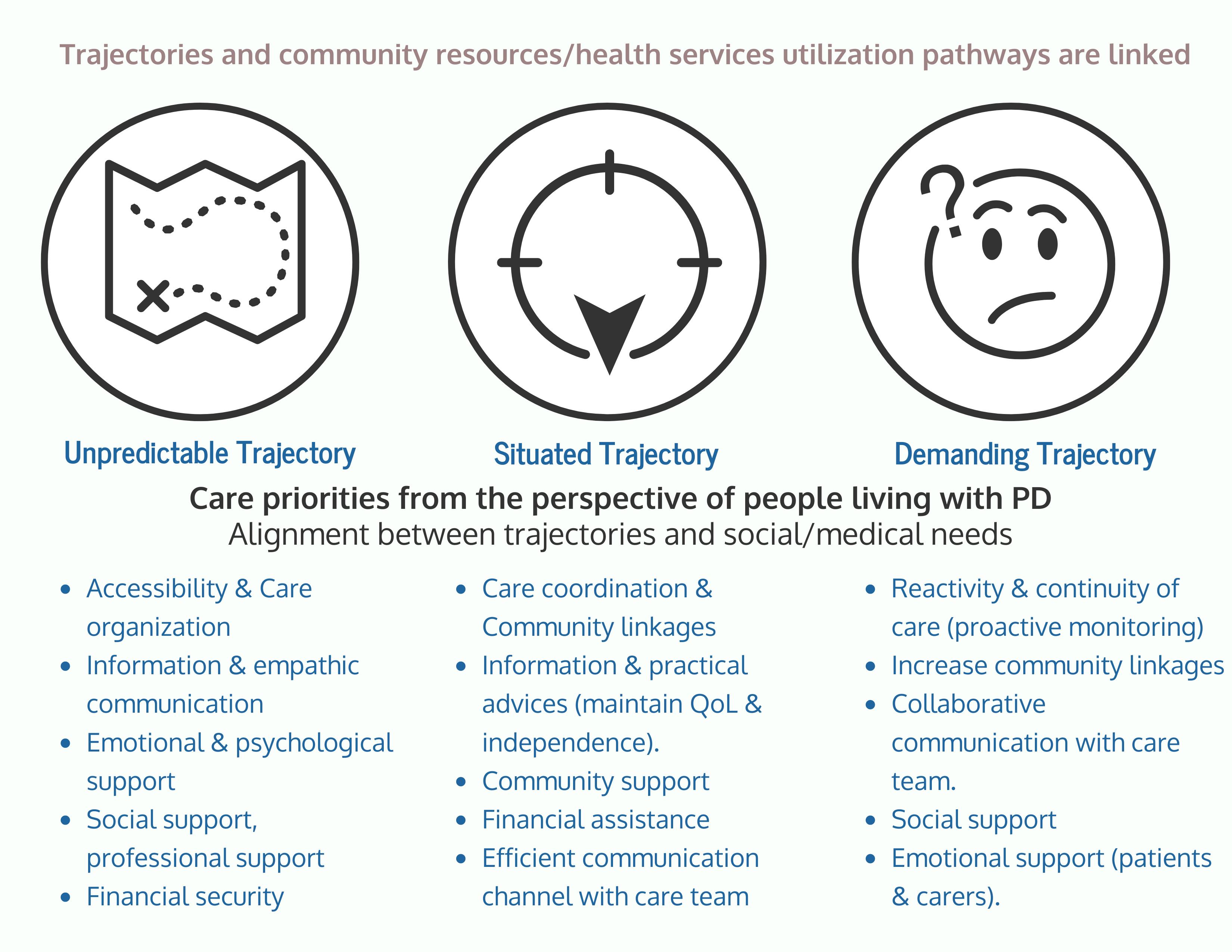

Supplement: Supplementary file 1 [file jpm-12-01001-s001.zip › Figure S5í¬Patientsí» trajectories identified in [22].jpg]
